# Novel Insights into Osteoclast Energy Metabolism

**DOI:** 10.1007/s11914-023-00825-3

**Published:** 2023-10-10

**Authors:** Maria G. Ledesma-Colunga, Vanessa Passin, Franziska Lademann, Lorenz C. Hofbauer, Martina Rauner

**Affiliations:** Department of Medicine III and Center for Healthy Aging, Technische Universität Dresden, 01307 Dresden, Germany

**Keywords:** Bioenergetics, Glycolysis, Oxidative phosphorylation, Osteoclasts, Mitochondria

## Abstract

**Purpose of Review:**

Osteoclasts are crucial for the dynamic remodeling of bone as they resorb old and damaged bone, making space for new bone. Metabolic reprogramming in these cells not only supports phenotypic changes, but also provides the necessary energy for their highly energy-consuming activity, bone resorption. In this review, we highlight recent developments in our understanding of the metabolic adaptations that influence osteoclast behavior and the overall remodeling of bone tissue.

**Recent Findings:**

Osteoclasts undergo metabolic reprogramming to meet the energy demands during their transition from precursor cells to fully mature bone-resorbing osteoclasts. Recent research has made considerable progress in pinpointing crucial metabolic adaptations and checkpoint proteins in this process. Notably, glucose metabolism, mitochondrial biogenesis, and oxidative respiration were identified as essential pathways involved in osteoclast differentiation, cytoskeletal organization, and resorptive activity. Furthermore, the interaction between these pathways and amino acid and lipid metabolism adds to the complexity of the process. These interconnected processes can function as diverse fuel sources or have independent regulatory effects, significantly influencing osteoclast function.

**Summary:**

Energy metabolism in osteoclasts involves various substrates and pathways to meet the energetic requirements of osteoclasts throughout their maturation stages. This understanding of osteoclast biology may provide valuable insights for modulating osteoclast activity during the pathogenesis of bone-related disorders and may pave the way for the development of innovative therapeutic strategies.

## Introduction

Bone is a dynamic tissue that underlies a fine-tuned balance between breaking down existing bone and building new bone. This so-called remodeling process is essential to maintain bone homeostasis and strength and to regulate calcium and phosphate metabolism [1]. Osteoclasts, specialized cells responsible for bone resorption, play a pivotal role in this process. Deficiency of osteoclast numbers or function results in elevated bone density, but increased fragility known as osteopetrosis, while overactive osteoclast function leads to reduced bone mass and susceptibility to fragility fractures, which is a hallmark of osteoporosis [2].

Recent research has focused on the activation of metabolic programs that take place in osteoclasts alongside the molecular mechanisms governing their differentiation and function. This emerging aspect in osteoclast biology, known as metabolic reprogramming, not only determines their complex phenotypic changes during differentiation ranging from single cells to large syncytia to polarized osteoclasts, but also enhances the process of bone resorption by providing essential energy resources [3, 4].

Osteoclasts originate from progenitor cells from the monocyte/macrophage lineage that undergo a process of differentiation and cell fusion to give rise to specialized, multinucleated, bone-resorbing polykaryons [5]. Recent investigations using lineage tracing and fate-mapping experiments have significantly contributed to our understanding of the intricate process behind osteoclast formation. During the developmental and neonatal stages, erythromyeloid progenitors emerge in the yolk sac and transform into colony-stimulating factor 1 receptor positive yolk sac macrophages, which serve as precursors for osteoclasts and play a vital role in creating space for postnatal bone marrow hematopoiesis [6•, 7•]. As postnatal life progresses, mononuclear monocytes derived from hematopoietic stem cells further contribute to the process of osteoclastogenesis. These hematopoietic stem cell–derived cells also fuse, in part, with long-lived erythromyeloid progenitors-derived osteoclasts, establishing a cohesive mechanism for the sustained presence and function of osteoclasts throughout adulthood [6•].

The activation and differentiation of osteoclasts are driven by two key factors: macrophage colony-stimulating factor (M-CSF) and the receptor activator of the nuclear factor kappa-B ligand (RANKL). M-CSF plays a crucial role in supporting the survival and proliferation of progenitor cells, while RANKL is important for the differentiation and maturation of osteoclasts. When RANKL binds to its receptor RANK on osteoclast precursor cells, it initiates a cascade of events that activates several transcription factors, including nuclear factor kappa-B (NF-κB), c-Fos/activator protein 1 (AP-1), and nuclear factor of activated T cells, cytoplasmic 1 (NFATc1) [3]. These transcription factors are critical for regulating gene expression, ensuring proper osteoclast differentiation, and maintaining their function.

Mature bone-resorbing osteoclasts attach to the bone surface and form a sealing zone, which allows them to release protons and proteolytic enzymes, including cathepsin K, into an isolated resorptive compartment, leading to the dissolution of the inorganic and degradation of the organic bone components under acidic conditions without affecting neighboring areas [8]. After bone resorption, osteoclasts retract and become quiescent, undergo apoptosis, or fission into osteomorphs, smaller motile cells that can circulate and fuse to re-form osteoclast in a process termed osteoclast recycling [9, 10•]. This recycling system of osteomorphs may contribute to energy efficiency and rapid osteoclast regeneration.

During their maturation, osteoclasts undergo various differentiation stages including precursor proliferation, the fusion of mononuclear cells, rearrangement of their cytoskeleton to produce polarized osteoclasts that can resorb bone, and eventually cell fission and fusion events that also require cytoskeletal rearrangements and new organization of nuclei and other organelles. These processes require energy that is provided via various sources including glucose, amino acids, and fatty acids at specific phases of differentiation [11, 12]. For instance, while several pathways including oxidative phosphorylation (OXPHOS), glycolysis, and the breakdown of lipids and amino acids are essential for providing energy during osteoclast differentiation, only glycolysis appears to be critical for bone resorption [2, 13, 14]. As certain metabolic pathways differentially affect the various stages of osteoclast maturation, research has been focusing on targeting pathways associated with metabolic reprogramming to identify their potential beneficial effects on pathological conditions. In this context, this review aims to investigate the latest discoveries regarding the influence of metabolic adaptations on osteoclast development. By gaining insights into how cellular metabolism influences the fate of osteoclasts, we can deepen our understanding of the intricate interplay between metabolism and bone remodeling. Moreover, these findings may have potential therapeutic implications for addressing various bone-related disorders.

## Mitochondria Bioenergetics

### Mitochondrial Biogenesis

**Fig. 1:**
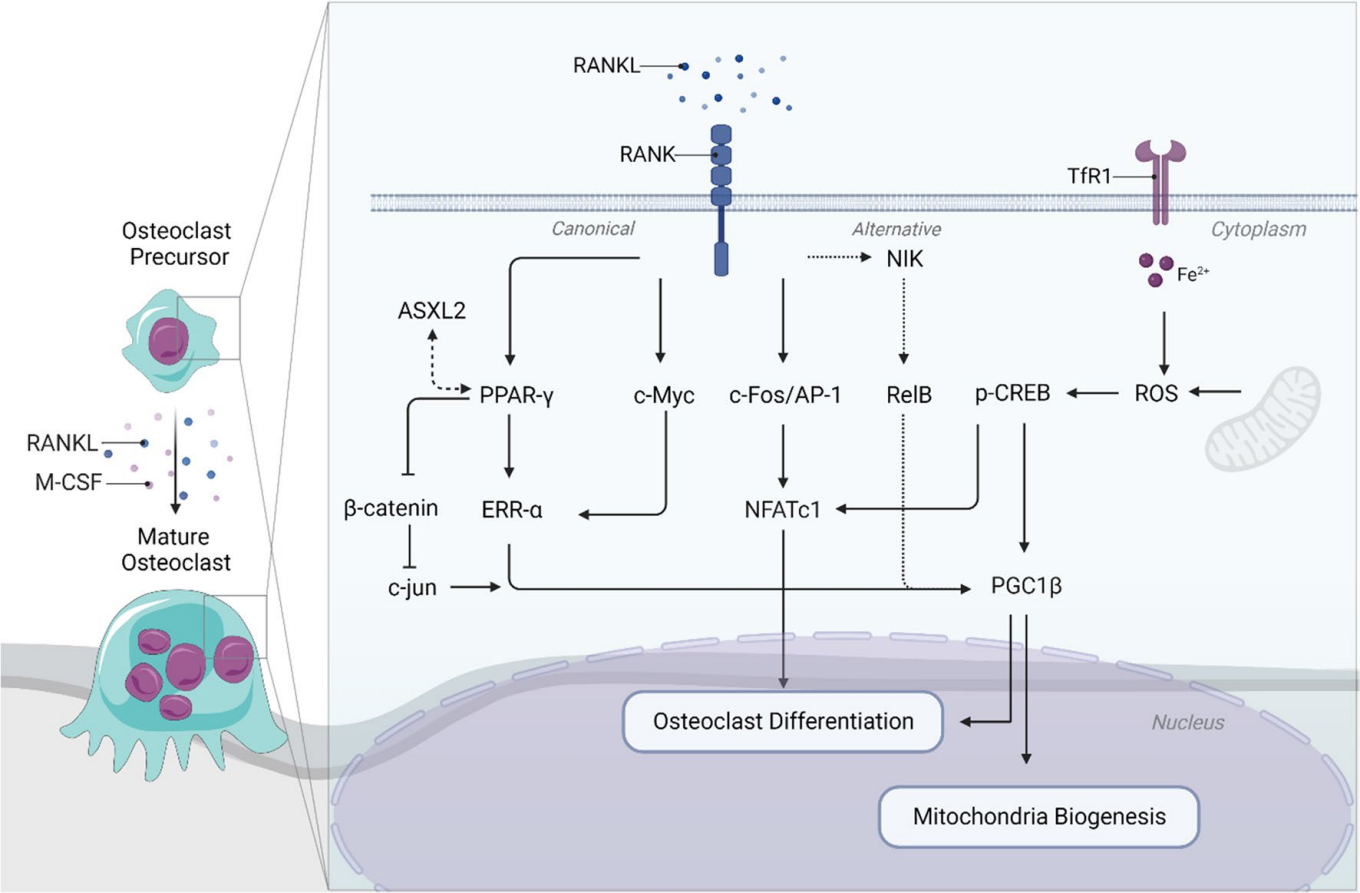
Overview of mitochondrial bioenergetics during osteoclast differentiation and function. Osteoclasts originate from monocyte/macrophage lineage cells, which merge to form specialized multinucleated cells. This process is regulated by M-CSF and RANKL. Upon RANKL signaling, a sequence of events is triggered, activating transcription factors like NFATC1. These factors regulate gene expression to ensure proper osteoclast differentiation and functionality. Given the high energy demands, metabolic adaptations are essential for the development and maturation of osteoclasts. This involves inducing mitochondrial biogenesis through both PGC1β-dependent and independent mechanisms, resulting in an increased number of mitochondria and robust OXPHOS activity to support osteoclastogenesis. *Created with Biorender*

Mitochondria are specialized organelles that play a crucial role in producing adenosine triphosphate (ATP), which is the primary source of energy transfer and storage [15]. As sites for aerobic respiration, these organelles take up glucose-derived pyruvate, amino acids, fatty acids, and other substrates to fuel the tricarboxylic acid (TCA) cycle. Through this process, reduced cofactors are generated, which then drive the electron transport chain (ETC) to ultimately produce up to 36 ATPs per one molecule of glucose via OXPHOS [16]. Studies conducted on osteoclasts derived from various species have consistently shown that these cells possess a high abundance of mitochondria [17–20]. While earlier reports suggested that osteoclasts inherit mitochondria from precursor cells [21], it has recently been revealed that RANKL-induced osteoclastogenesis also results in a significant increase in mitochondria biogenesis [22]. Several factors related to mitochondrial biogenesis and function, including peroxisome proliferator–activated receptor-gamma coactivator-1 β (PGC1β), peroxisome proliferator–activated receptor gamma (PPAR-γ), and estrogen-related receptor α (ERRα) play a fundamental role in osteoclast development and function (Fig. 1).

In particular, PGC1β is a critical regulator of mitochondrial biogenesis and function in osteoclasts [22]. Knockout studies have suggested its significance in osteoclast differentiation as its absence inhibited in vitro osteoclastogenesis and led to increased bone mass due to impaired osteoclast function [22, 23]. Mechanistically, the induction of PGC1β by RANKL leads to an increased demand for iron uptake through the transferrin receptor 1 (TfR1) and its subsequent delivery to mitochondria. This process is essential for inducing oxidative respiration, ensuring an adequate energy supply for the osteoclasts. Furthermore, phosphorylation of cyclic AMP response element (pCREB) contributes to the induction of PGC1β transcription through reactive oxygen species (ROS), thereby expediting the process of osteoclastogenesis [22]. Moreover, iron homeostasis has a significant impact on osteoclastogenesis. Deletion of TfR1 in myeloid precursor cells and mature osteoclasts or iron chelation leading to intracellular iron deficiency results in increased bone mass due to impaired osteoclast differentiation [24, 25]. Conversely, the deletion of the iron exporter ferroportin in murine myeloid cells leads to increased iron accumulation and stimulates osteoclastogenesis both in vitro and in vivo [26]. Furthermore, studies comparing mice with targeted removal of *Pgc1b* in myeloid cells indicate that PGC1β does not influence osteoclast differentiation in vitro or in vivo, as mice lacking *Pgc1b* exhibit increased bone mass. This finding suggests that PGC1β impacts bone resorption by halting osteoclast function rather than affecting their differentiation [27•]. The inconsistencies in these findings may potentially be attributed to differences in the gene targeting methods employed, such as the use of distinct cre-lines like Tie2 vs. Lysm, which may result in varying effects.

PGC1β can also be induced by activating PPAR-γ. Loss of PPAR-γ function in hematopoietic cells hinders osteoclast differentiation, resulting in increased bone mass. Conversely, when PPAR-γ is activated, for example, with the antidiabetic drug rosiglitazone, it indirectly promotes PGC1β expression by reducing β-catenin protein levels and suppressing c-Jun expression. This, in turn, promotes osteoclast differentiation [23]. Additionally, PPAR-γ induces ERRα expression and, along with PGC1β, activates mitochondrial genes for OXPHOS, boosting mitochondrial biogenesis and supporting osteoclast function [23]. PPAR-γ also interacts with ASXL transcriptional regulator 2, promoting osteoclast formation and mitochondrial biogenesis through c-Fos/NFATc1 induction and PGC1β activation [28].

Besides these pathways, there is an alternative downstream pathway of RANKL that involves the activation of NF-κB-inducing kinase (NIK) and RelB, which modulates PGC1β activation. Cells lacking alternative NF-κB pathway activation (NIK and RelB ablation) exhibit defects in mitochondrial biogenesis, displaying poor induction of PGC1β and impaired osteoclast differentiation [29]. Interestingly, overexpression of PGC1β in RelB and ASXL2-deficient cells rescues mitochondrial biogenesis but fails to restore impaired osteoclastogenesis. Conversely, overexpression of NFATc1 fully restores the differentiation process, yet it does not rescue bone resorption [28, 29]. These results suggest that mitochondrial biogenesis and osteoclast differentiation are independently regulated and involve both PGC1β-dependent and PGC1β-independent pathways (Fig. 1).

### Oxidative Respiration

**Fig. 2:**
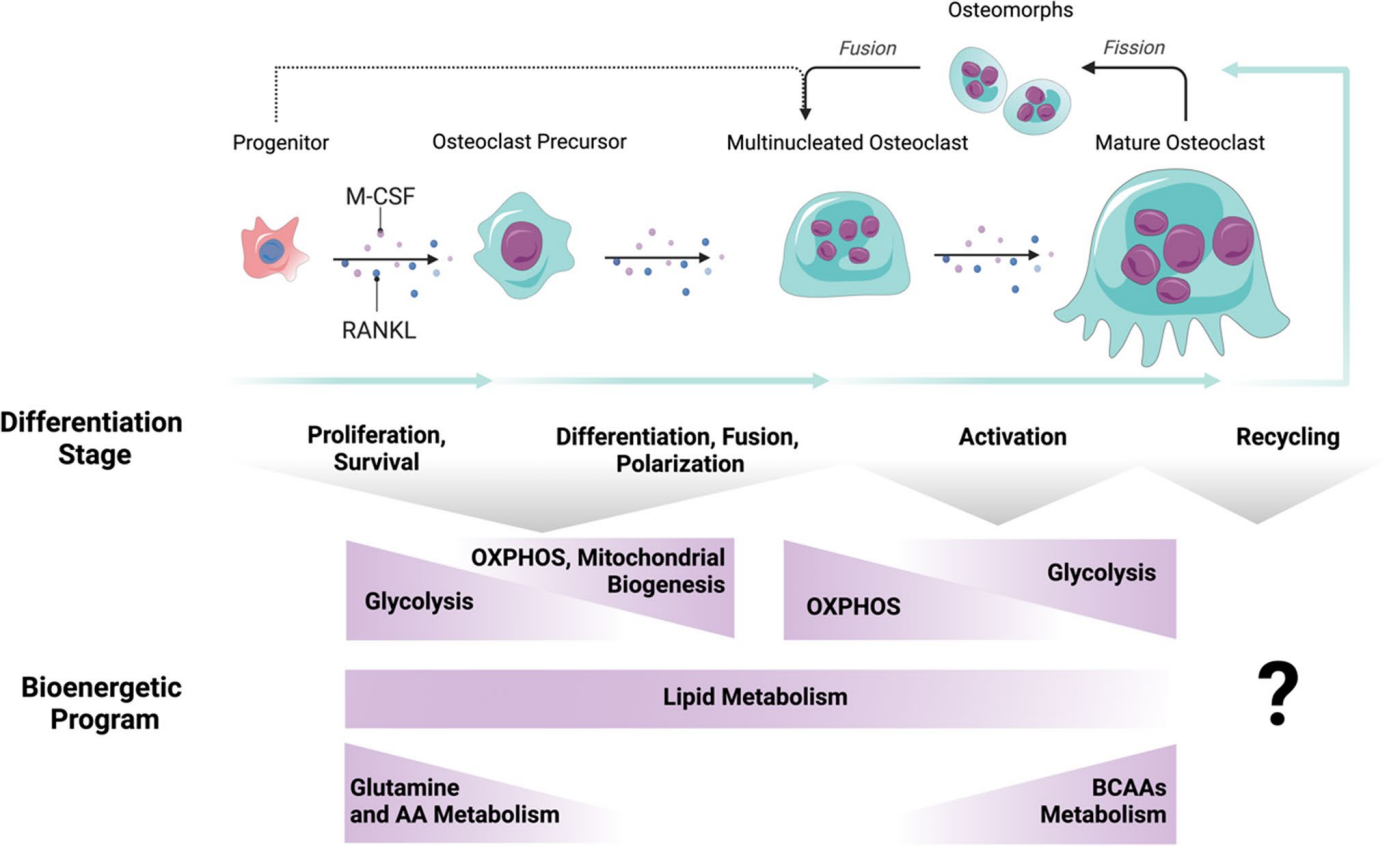
How energy metabolism contributes to osteoclast differentiation and function. Energy metabolism plays a crucial role in osteoclast differentiation and function, involving several pathways such as oxidative phosphorylation (OXPHOS), glycolysis, lipid metabolism, glutamine, amino acids (AA), and branched-chain amino acids (BCAAs) metabolism. These pathways are essential for providing the energy needed during the processes of osteoclast differentiation and functional activation. Additionally, both lipid metabolism and amino acid uptake contribute as energy pathways throughout all stages of osteoclast development and function, while the metabolic aspects of the osteomorphs are yet not been fully explored. *Created with Biorender*

Mitochondrial abundance and robust OXPHOS activity are closely linked to the process of osteoclastogenesis (Fig. 2). While it remains uncertain whether mitochondrial biogenesis is a prerequisite for the development and ultimate activity of osteoclasts, it is now acknowledged that as osteoclast differentiation advances, there is a notable increase in the protein expression of OXPHOS complexes. This leads to enhanced oxygen consumption, coinciding with a simultaneous decrease in glycolysis. These changes collectively represent an active metabolic reprogramming aimed at augmenting energy production to support osteoclast function [19].

RANKL-inducible c-Myc is a transcriptional factor that promotes osteoclast differentiation, primarily by influencing oxidative metabolism. c-Myc directly regulates NFATc1 and also plays a role in inducing ERRα. Consequently, c-Myc regulates alternative pathways that are required to cooperate with NFATc1 to promote osteoclastogenesis, while ERRα governs the expression of genes related to the ETC and OXPHOS. Ablation of c-Myc in osteoclast precursor cells results in defective osteoclast development and an associated increase in bone mass. Notably, the impact of c-Myc is independent of mitochondrial biogenesis, as overexpression of c-Myc does not affect PGC1β regulation [30], which suggests the uncoupling of PGC1β from OXPHOS in osteoclastogenesis.

Furthermore, the significance of OXPHOS in osteoclastogenesis has been investigated through various approaches, including inhibition of ETC complexes and disruption of the mitochondrial network. Lack of *Ndusf4* (Complex I subunit) or blockade of Complex I activity using rotenone in precursor cells inhibits osteoclast differentiation and the formation of resorption pits in vitro [31]. In line with these findings, *Pgc1b* deficiency in osteoclasts dampens OXPHOS function consequently impairing cytoskeleton organization and bone resorptive activity [27•]. Inducing mitochondrial dysfunction by deleting *Mfn1* and *Mfn2*, *Drp1*, or *Opa1* also leads to decreased osteoclastogenesis by impairing signaling to NFATc1, suggesting that the primary impact of increased mitochondrial biogenesis during osteoclast formation may not be related to ATP production [32–34]. Consistent with this, deleting the mitochondrial transcription factor A (*Tfam*) in mature osteoclasts results in a significant reduction in intracellular ATP levels and accelerated apoptosis, but it paradoxically increases bone resorption activity [20]. Interestingly, mature osteoclasts have been described as ATP-depleted cells compared to less differentiated cells, exhibiting lower levels of intracellular ATP and fewer copies of mtDNA, despite having a higher number of mitochondria [35]. Conversely, high concentrations of ATP inhibit osteoclast formation and activity. These observations suggest that reducing OXPHOS (and consequently ATP levels) may enhance their resorption activity, and this function may involve a metabolic switch toward glycolysis.

Altogether, these findings emphasize the role of OXPHOS as a predominant bioenergetic pathway during osteoclast differentiation, although it is not essential for the ultimate resorption function of mature osteoclasts. Additionally, the activation of PGC1β to regulate mitochondrial biogenesis suggests the involvement of several downstream factors and implies that the stimulation of new mitochondria is not a mandatory requirement for osteoclast formation and function.

## Glycolysis as a Relay in Osteoclast Function

Glucose is the major source of energy for bone growth and development and in particular, also mature osteoclasts prefer glucose as their principal nutrient [12, 13, 36] (Fig. 2). First studies using chicken osteoclasts showed that instead of fatty acids or ketone bodies, glucose and to a lesser extent lactate stimulated bone degradation and that the glucose uptake rate of mature osteoclasts almost doubled when they were attached to the bone in vitro [36, 37]. Vice versa, glucose deprivation or blocking glucose uptake inhibits the resorptive activity of murine osteoclasts [4, 36].

Glycolysis, a metabolic pathway taking place in the cytosol, breaks down glucose to provide the cell with a steady supply of energy in the form of ATP. A series of reactions performed by several tightly controlled glycolytic enzymes convert glucose to two pyruvate molecules to generate two ATP and two reducing equivalents in the form of nicotinamide adenine dinucleotide (NADH) [38]. In the process of aerobic glycolysis, lactate is produced from glucose instead of pyruvate even under oxygen-rich conditions to regenerate NAD + necessary for further glycolysis [13]. Several glycolytic intermediates serve as precursors for biosynthetic pathways such as the TCA cycle, pentose phosphate pathway, or glycerol pathway, and thus, glycolysis is a key element in the metabolism of all mammalian cells including osteoclasts [4, 12, 13, 19, 21, 36, 38].

The uptake of glucose through the plasma membrane is facilitated by glucose transporters (GLUT) and the expression of GLUT1 and GLUT3 encoded by the genes *Slc2a1* and *Slc2a3*, respectively, has been reported for murine osteoclasts and the osteoclast-like cell line RAW264.7 [4, 36, 39]. In mouse osteoclasts, expression of GLUT1 and glycolytic genes such as hexokinase (HK), phosphofructokinase (PFK), and pyruvate kinase (PKM, muscle type), rate-limiting enzymes of glycolysis, as well as lactate dehydrogenase (LDH) A and B increase progressively during RANKL-induced osteoclastogenesis [4]. In accordance, lactate production, glucose consumption, and their ratio defining the glycolytic efficiency increase toward mature stages of mouse osteoclast differentiation [4, 19]. This metabolic adaption of enhanced aerobic glycolysis together with elevated glutamine usage is orchestrated by the transcription factor hypoxia‐inducible factor 1‐alpha (HIF-1α) and c‐Myc and further relies on a balanced interaction of nutrient and energy sensors such as the mammalian target of rapamycin (mTOR) and the adenosine monophosphate (AMP)–activated protein kinase (AMPK) [4]. Nevertheless, both glycolytic and oxidative metabolism must be activated during mouse osteoclast differentiation for efficient energy and intermediate production from glucose taken up mainly by GLUT1 [4, 36, 39].

In contrast to rodent models, a study using human osteoclast cultures showed that osteoclast differentiation relies on enhanced OXPHOS while glycolytic efficiency decreased [19]. Nevertheless, osteoclast activity and in particular, bone matrix resorption required glycolysis, and associated enzymes are localized close to the sealing zone where the high energy-consuming activities occur [19]. A recent RNA-seq study using mononuclear cell–derived osteoclasts from the peripheral blood of healthy donors showed also an enrichment of genes involved in glycolysis and gluconeogenesis, besides OXPHOS and citrate cycle, as compared to their precursors obtained from the same donor [40], indicating that both differentiation and activity of human osteoclasts might be fueled by glycolytic processes, at least to certain extents.

In addition, glycolytic intermediates or products themselves have been shown to affect osteoclast differentiation and activity. As such, fructose 1,6-bisphosphate has been reported to suppress RANKL-induced osteoclastogenesis and TRAP activity through NF-kB/NFATc1 pathway inhibition in bone-marrow-derived murine osteoclasts when given in high concentrations (100 µM, 300 µM) in vitro [41]. Furthermore, lactate production progressively increases during osteoclast development, and correspondingly, the expression of LDH, which catalyzes the reaction of pyruvate into lactate, is elevated in mature mouse bone marrow-derived osteoclasts [42]. Loss of the LDHA or LDHB subunit suppresses both glycolysis and mitochondrial respiration and leads to less RANKL-induced osteoclast formation caused by impaired precursor fusion [42]. In support of the importance of aerobic glycolysis and lactate production in actively resorbing osteoclasts, a recent study showed that blocking glycolysis with 2-deoxy-glucose (2DG) suppressed bone resorption which could be rescued by combined lactate and pyruvate supplementation while the metabolites alone had no effect [43•].

Given the impact of aerobic glycolysis on osteoclast biology in vitro, recent preclinical research aims to identify new therapeutic strategies for osteoclast-driven bone diseases by modulating osteoclastic glucose metabolism. In a mouse model of postmenopausal osteoporosis, systemic administration of 2DG to block glycolysis or LDHA inhibitor GSK2837808A to interfere with lactate production mitigated bone loss in ovariectomized mice as compared to sham-operated mice [43•]. Moreover, 6-phosphofructo-2-kinase/fructose-2,6-bisphosphatase 3 (PFKFB3), a crucial regulator of glycolysis, is upregulated during osteoclast formation and genetic/pharmacological PFKFB3 inhibition impaired osteoclast function and development [44]. In line, in vivo treatment with PFK15, a PFKFB3 inhibitor, prevented bone loss in ovariectomized mice by reducing bone resorption [44]. Another critical modulator of aerobic glycolysis, pyruvate dehydrogenase kinase 2 (PDK2), is upregulated during osteoclastogenesis and either knockout or pharmacological blockade of PDK2 delayed bone loss in ovariectomized mice potentially by inhibition of the RANKL/NFATc1 pathway [45].

Taken together, these findings imply that mature osteoclasts heavily rely on aerobic glycolysis for bone resorption, while osteoclast formation requires both glycolytic and oxidative metabolism to fulfill the high energetic requirements. Thus, interfering with aerobic glycolysis in osteoclasts might represent a potential therapeutic strategy for osteoporosis treatment to inhibit bone resorption, while keeping osteoclastogenesis and potentially coupling to osteoblasts intact [43•].

## Lipid Metabolism

Lipids are essential building blocks of biological membranes, and signal transducers but are also important for energy supply and storage. They exist as various species. The human bone marrow niche contains triglycerides, cholesterol, free fatty acids, and phospholipids [46]. Oxidative metabolism of lipids yields more ATP compared to other substrates (e.g., glucose) and generates the reducing equivalents NADH and FADH_2_ important for the generation of ATP via the ETC [47]. Lipid accumulation is associated with changes in bone mass. Various studies link hyperlipidemia as well as hypercholesterolemia to decreased bone mass and increased osteoclastic bone resorption [48–50].

Fatty acids (FA) can be obtained by de novo lipid synthesis or uptake via specified proteins. Beta-oxidation of FA in the mitochondria is the major pathway for FA degradation. Therefore, activation of FA by conjugation to coenzyme A is followed by transport to mitochondria mediated by carnitine palmitoyltransferases (CPT). CPT1 located on the outer mitochondrial membrane generates acyl-carnitine which allows the transport across the mitochondrial matrix via the carnitine-acylcarnitine transferase. CPT2 located at the inner mitochondrial membrane completes the process by reconverting the acyl-carnitine into acyl-CoA. During β-oxidation inside the mitochondria, acyl-CoAs are degraded in a stepwise shortening to release acetyl-CoA that can enter the TCA cycle [51]. In case of excess, FA can also be stored in lipid droplets as triglyceride or cholesterol ester. Oxidation of FA increases during osteoclast differentiation and is required for normal osteoclast formation in vitro [52•, 53, 54]. Blocking β-oxidation of long-chain FA by ablation of *Cpt2* in myeloid cells leads to an increase in trabecular bone volume in female mice with reduced numbers of osteoclasts in vivo [52•]. In line, the activation of CPT1a-mediated FA oxidation present in patients with rheumatoid arthritis increases osteoclast precursor fusion [55]. FA are classified based on the number and position of double bonds. Various FA species have shown distinct effects on osteoclasts (reviewed in [56, 57]). For instance, the saturated FA palmitic acid was found to enhance osteoclastogenesis, even in the absence of RANK [54], and the addition of long-chain poly-unsaturated FA exerts negative effects on osteoclast viability, differentiation, and maturation [58–60]. On another note, short-chain fatty acids and other microbial metabolites produced through the fermentation of dietary fibers in the gut have been observed to suppress osteoclastogenesis both in vitro and in vivo [61]. Moreover, saturated FA have been found to promote osteoclastogenesis by preventing apoptosis [62].

Cholesterol is a key component of biological membranes defining their structural and functional characteristics [63]. It can be supplied by the uptake of lipoproteins or synthesized via the mevalonate pathway starting with acetyl-CoA. Osteoclasts have been shown to rely on cholesterol uptake rather than de novo synthesis [64]. The uptake of low-density lipoprotein (LDL) cholesterol is essential for osteoclastogenesis [65, 66]. LDL receptor knockout mice show increased bone mass due to decreased osteoclast formation resulting in impaired fusion of osteoclast precursors [67]. Knock-down of the master transcription factor of cholesterol homeostasis *Srebf2* in osteoclasts negatively influences their differentiation [68]. One possible mechanism suggested by the authors is that SREBP2 controls osteoclast differentiation through LDL receptor gene expression and cholesterol uptake. Additionally, cholesterol represents a constituent of lipid rafts, which are signaling centers mediating membrane trafficking, endocytosis, and signal transduction that are involved in RANK-RANKL signaling during osteoclastogenesis [69].

The mevalonate pathway is a key pathway that is required for the biosynthesis of cholesterol and for the post-translational modifications of signaling proteins. Two large classes of drugs inhibit this pathway with differing effects: while statins inhibit the 3-hydroxy-3-methyl-glutaryl-coenzyme A reductase and thereby lead to an effective reduction of cholesterol, bisphosphonates target the farnesyl diphosphate synthase, thereby disrupting the prenylation of small GTPase proteins [57]. Due to their biochemical similarity to pyrophosphate, bisphosphonates accumulate in the bone tissue and thus, have a particularly strong effect on inhibiting osteoclasts, which ingest the bisphosphonates bound to within the bone tissue. Consequently, BPs are commonly employed in clinical settings to treat conditions characterized by excessive bone resorption [70].

Altogether, these studies highlight the significance of lipid metabolism in osteoclasts, the differential role of various lipid species on osteoclast activity and suggest lipids as potential targets for therapeutic intervention in pathological bone loss.

## Amino Acid Metabolism

Despite the well-established understanding that dietary sufficient protein intake is closely associated with bone health, and osteoclasts expressing amino acid transporters [71], only a few studies have examined the significance of amino acid metabolism in osteoclast differentiation and function (reviewed in Da et al. [2] and Devignes et al. [14]).

Amino acids classified as ketogenic or glucogenic are crucial for protein synthesis, but can also function as an alternative energy source by undergoing metabolism through the TCA cycle [71]. Glutamine metabolism, in particular, has been found to play an important role in osteoclast differentiation (Fig. 2). The primary sustenance of glutamine metabolism in these cells is facilitated through its uptake via the SLC1A5 transporter. Inhibition of this transporter, either through genetic manipulation or pharmacological intervention, leads to a decline in intracellular glutamine levels and hampers osteoclastogenesis in vitro [4, 72•]*.* As glutamine is broken down to form α-ketoglutarate (α-KG), which can be taken up by the TCA cycle, supplementing the cells with a cell-permeable form of α-KG effectively restores the phenotypic defects caused by inhibiting the transporter [4]. Furthermore, it has been observed that the coordination of HIF-1α and c-Myc expression stimulates glutamine consumption by osteoclasts and is dependent on a balanced regulation of the nutrient and energy sensors, mTOR, and AMPK activities. Inhibition of c-Myc leads to impaired osteoclast differentiation by affecting the function of SLC1A5 and glutaminase [4]*.* Moreover, in vitro studies have emphasized the significance of glutamine transport in osteoclasts. The absence of l-glutamine, a component of cell culture media, leads to defective osteoclastogenesis, which can be restored in a dose-dependent manner by its addition. Notably, an increase in glutamine uptake primarily promotes biosynthesis without affecting ATP production through any pathway [4, 73].

Recent studies have emphasized the crucial role of arginine in RANKL-induced osteoclastogenesis. While the uptake of arginine remains unchanged after RANKL treatment, extracellular arginine supports the activity of the TCA cycle and OXPHOS [74]. In studies using ^13^C-labeled tracers, it has been observed that arginine, involved in the urea cycle, is metabolized to form fumarate in the TCA cycle, contributing to the energy supply. Depletion of extracellular arginine, through its conversion to ornithine and urea, reverses the accumulation of metabolic intermediates induced by RANKL, reduces the expression of TCA cycle enzymes, and upregulates enzymes involved in serine and purine biosynthesis [75]. Interestingly, the addition of α-KG does not rescue the defect in osteoclastogenesis caused by arginine depletion, suggesting the involvement of other arginine derivatives [74].

The essential amino acid methionine also regulates osteoclast differentiation and function. RANKL induces an increased production of S-adenosylmethionine, which mediates DNA methylation through DNMT3a resulting in epigenetic transcriptional repression of anti-osteoclastogenic genes that benefit osteoclast differentiation [76]. Furthermore, a diet rich in methionine has been shown to elevate homocysteine levels, which consequently causes oxidative stress and promotes bone loss through increased bone resorption by osteoclasts [77].

In a similar vein, when studying the effects of adding a combination of aromatic amino acids, namely phenylalanine, tyrosine, and tryptophan, there is evidence supporting its promotion of osteoclast resorption activity. However, mRNA expression analysis indicates that this same combination reduces the expression of early and late osteoclast differentiation markers [78, 79]. Moreover, observations on the intermediate metabolites of the amino acids leucine, isoleucine, and phenylalanine, have been reported to contribute to osteoclast differentiation by entering the TCA cycle as acetyl-CoA thereby regulating ATP supply and countering the inhibitory effect caused by the lack of parent amino acids on osteoclastogenesis [75]. These findings suggest that amino acid uptake and metabolism may affect different stages of osteoclast differentiation.

In a related context, recent studies have highlighted the significance of branched-chain amino acids (BCAAs), namely, leucine, isoleucine, and valine, since their levels and uptake increase during osteoclasts metabolism. Their withdrawal severely hampers osteoclast maturation without affecting earlier differentiation stages. Branch-chain aminotransferase 1 (BCAT1) is the enzyme responsible for metabolizing BCAAs, transferring the amine group onto α-KG, thereby producing glutamate. When BCAT1 is genetically removed, osteoclastogenesis is impaired, leading to increased bone mass without impacting osteoblasts. In line with these findings, gabapentin-mediated BCAT1 inhibition also disrupts NFATc1 expression in pre-osteoclasts, affecting their fusion [80•]. Furthermore, the metabolism of BCAAs in osteoclasts is controlled through their transporter SLC7A5 (or LAT1) and involves downstream effects on mTOR. When *Slc7a5* is specifically deleted in osteoclasts, mTOR signaling is impaired resulting in an opposite effect where bone resorption is enhanced [81]. This particular phenotype can be corrected by the genetic activation of mTOR, suggesting that the LAT1-mTORC1 axis controls NFATc1 nuclear accumulation and expression through Akt-glycogen synthase kinase 3β (GSK3β) axis and the canonical NF-κB pathway, respectively.

Taken together, these findings underscore the significance of amino acid uptake and utilization during osteoclast differentiation. However, additional research is necessary to gain a comprehensive understanding of the complex interplay between uptake and metabolism and its ultimate impact on osteoclast behavior.

## Conclusions

Metabolic reprogramming in osteoclast biology is a crucial and emerging area of research, shedding light on the fundamental processes that control bone remodeling and skeletal homeostasis. Despite efforts to explore single aspects, the field still lacks comprehensive characterization. Unlike other cell types, osteoclast differentiation does not follow a defined phenotype; instead, the cellular metabolism must continuously adjust to the changing needs during differentiation and cellular actions. Thus, understanding this intricate relationship between metabolic pathways and key regulators of osteoclastogenesis might offer great potential for developing targeted therapeutic approaches to manage bone-related disorders and improve overall bone health.
